# Preoperative radiation in large angiomatosis of the breast, attempting breast conserving surgery: multidisciplinary approach

**DOI:** 10.1093/jscr/rjy024

**Published:** 2018-02-14

**Authors:** A Chulakadabba, S Denariyakoon, P Chakkabat, K Shotelersuk, P Sampatanukul, D Boonjunwetwat, K Chatamra

**Affiliations:** 1 Queen Sirikit Centre for Breast Cancer, Thai Red Cross Society, Bangkok, Thailand; 2 King Chulalongkorn Memorial Hospital, Thai Red Cross Society, Bangkok, Thailand; 3 Division of Therapeutic Radiology and Oncology, Department of Radiology, Faculty of Medicine, Chulalongkorn University, Bangkok, Thailand

## Abstract

Angiomatosis of the breast is very rare. The presentations are including breast mass, skin discoloration and breast enlargement that mimic to angiosarcoma. The imaging could suggest non-specific vascular tumors. The histology should be obtained for the certain diagnosis. Surgical excision is the standard treatment. To our knowledge, this is the first case that preoperative radiation is given. Currently, radiation is occasionally used in benign condition. This case shows the successful result of preoperative radiation for achieving breast conserving surgery in large angiomatosis of the breast.

## CASE REPORT

**Figure 1:**
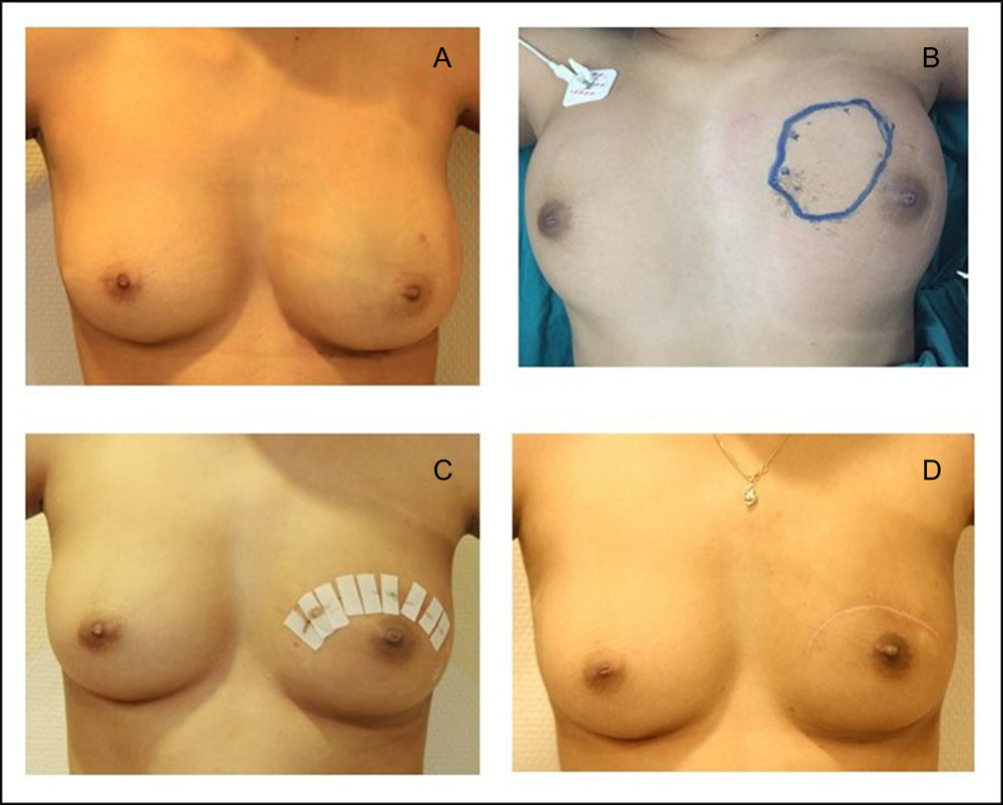
(**A**) Asymmetrical enlargement of left breast with tumour occupied entire left breast. (**B**) Post radiation, the ultrasound skin mark located the demonstrable tumour. (**C**) Post wide excision Day 7. (**D**) A post wide excision 6 months.

**Figure 2:**
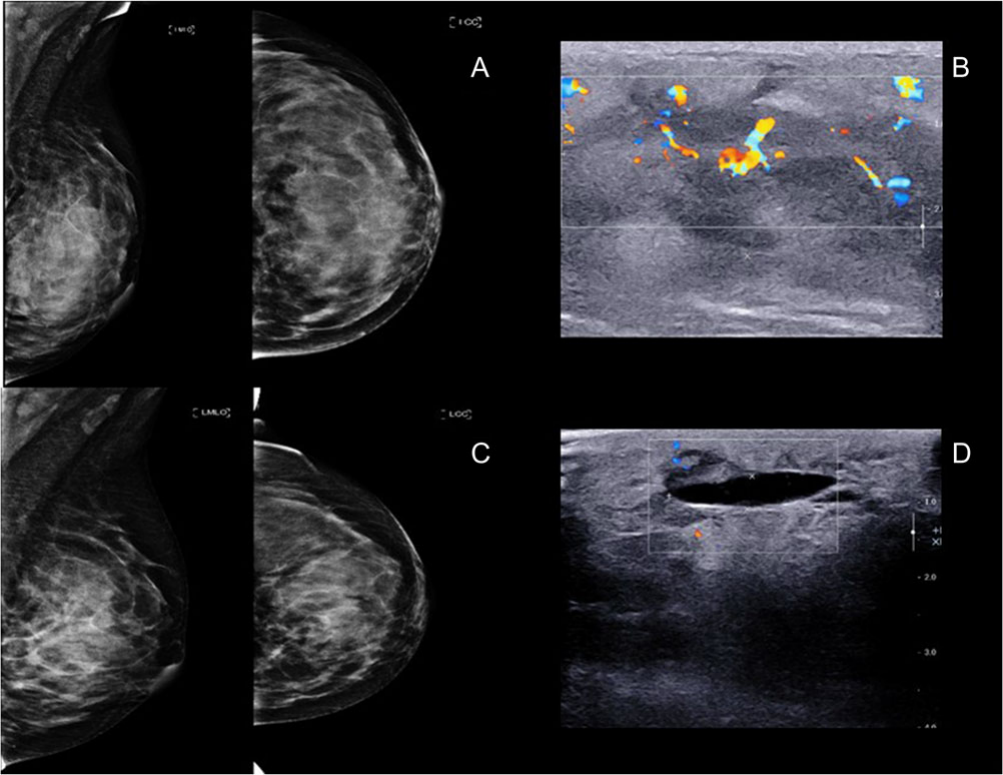
(**A** and **B**) Mammogram and ultrasound showed an ill-defined big mass at left mid inner quadrant with hypervascularity, subdermal edema and overlying skin thickening. (**C** and **D**) Mammogram and ultrasound post radiation showed 5.4 × 2.4 cm^2^. Ill-defined mass at left upper to mid inner quadrant with interval decreased internal hypervascularity.

A 24-year-old female came with a history of asymmetrical enlargement of left breast for 4 months. The physical examination revealed asymmetrical enlargement of left breast with a large ill-defined lump mainly occupied in the upper inner quadrant. No skin discoloration or ulcer was seen (Fig. 1). Digital mammography with tomosynthesis (Hologic Selenia Dimensions, MA, USA) demonstrated extremely dense breasts with asymmetrical enlargement of the left breast. There was a large ill-defined mass mainly occupying at upper inner quadrant. No associated architectural distortion or suspicious microcalcifications were seen. Additional ultrasonography showed an ill-defined infiltrative mixed hypoechoic and hyperechoic mass at the same area. This mass demonstrated multiple vascular structures and a large arterial feeder. There were few benign left axillary lymph nodes (Fig. 2).

**Figure 3:**
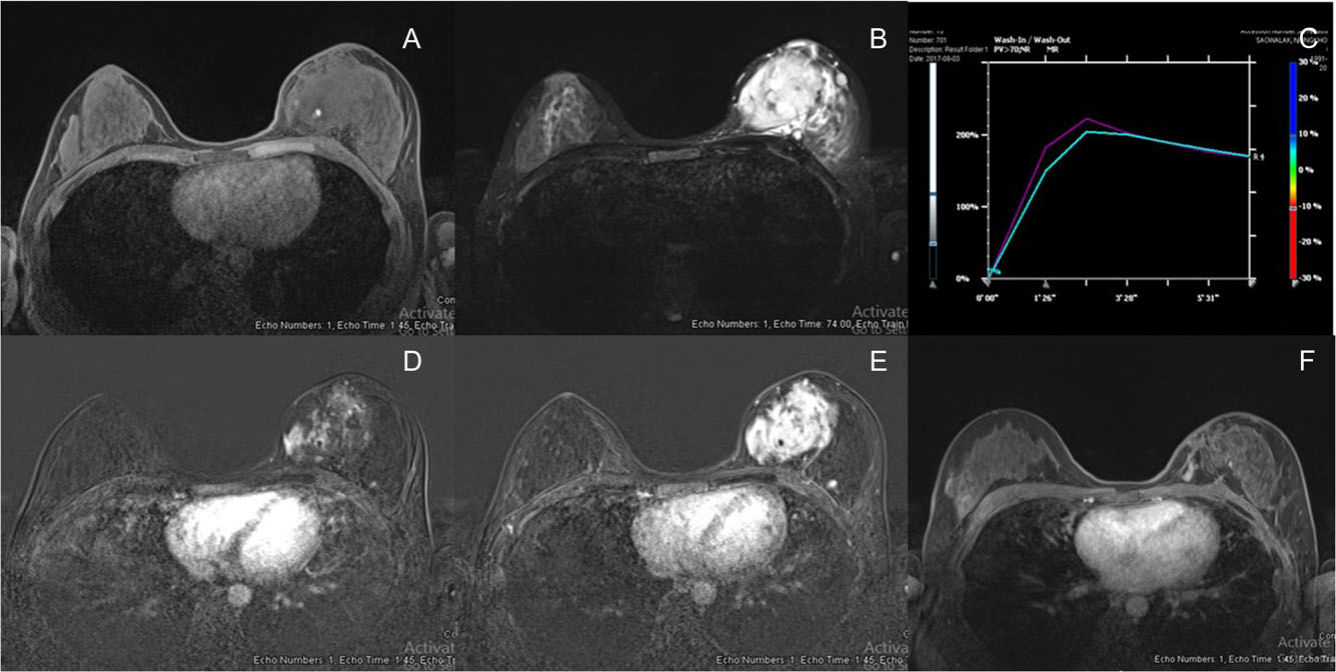
(**A**) Breast MRI showed isosignal intensity in T1WI with hemorrhagic spot (arrow). (**B**) Breast MRI showed strongly high signal intensity in T2WI. (**C**) Signal time curve showed type 2 and type 3 kinetic curve pattern but type 2 in majority. (**D**) Breast MRI showed rapid arterial enhancement in early arterial phase. (**E**) Breast MRI showed mixed plateau and washout enhancement in delayed phase. (**F**) Breast MRI post radiation showed interval decreased in size, currently showed slow arterial enhancement with persistent enhancement on delayed phases (type I kinetic).

Breast MRI (1.5 T, Espree, Siemens) was performed. It displayed a large infiltrative mass with indistinct margin, approximated ~8.9 × 7.8 × 5.5 cm^3^. The mass showed isosignal intensity on T1WI and strongly hypersignal intensity on T2WI. After intravenous contrast administration, this mass demonstrated rapid arterial enhancement with mixed plateau and washout on delayed phase (types II and III kinetic curve enhancement pattern). Some areas of the mass showed washout enhancement pattern on delayed phases (type III kinetic curve pattern) but the majority of the mass showed plateau enhancement. This mass extended anteriorly close to the skin without evidence of skin invasion or skin nodules. The mass also contained few small internal hemorrhagic foci (Fig. 3).

Repeated ultrasound-guided biopsy was performed. The histomorphology showed atypical vascular proliferation that was not correspondent for angiosarcoma. The CD31, CD34, FLI-1 and Factor VIII were positive, while S-100 and AE1/AE3 were negative. The immunohistochemical study supported endothelial nature, However, the entire specimen was required for accurate result.

Preoperative radiation was suggested by our multidisciplinary team. After the external beam irradiation with total dose of 60 Gy in 30 fractions was obtained, the physical examination showed markedly decreased size of the tumour. Follow up mammogram and ultrasound demonstrated an ill-defined mass with interval decreased in size and vascularity. Correspondingly, the follow up MRI showed interval decrease in size and enhancement of a 5.3 × 3.0 cm^2^. ill-defined infiltrative enhancing mass at left upper inner quadrant. This mass showed slow arterial enhancement with persistent enhancement on delayed phase (type I kinetic curve enhancement pattern) (Fig. 3). The patient had mild erythematous skin after radiation. As a result, breast conserving surgery was suggested by our multidisciplinary team.

**Figure 4:**
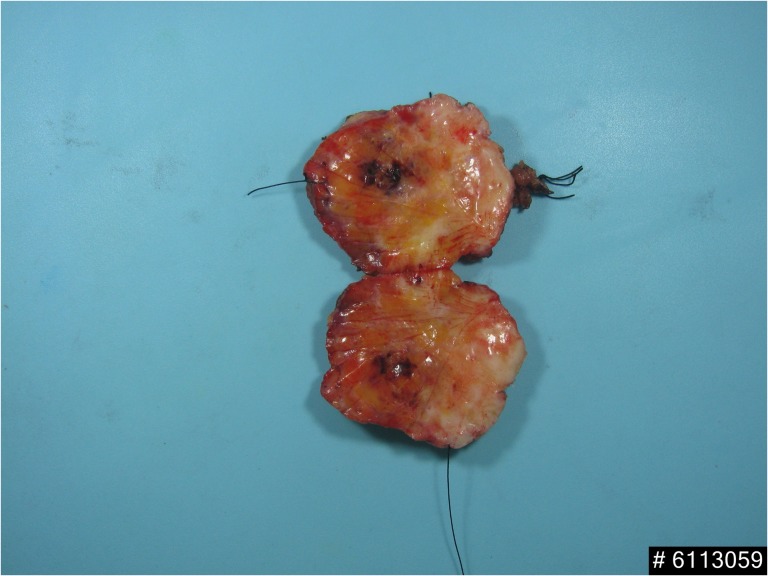
Wide excision specimen showed area of congested red pigmented tissue with internal hemorrhage.

**Figure 5:**
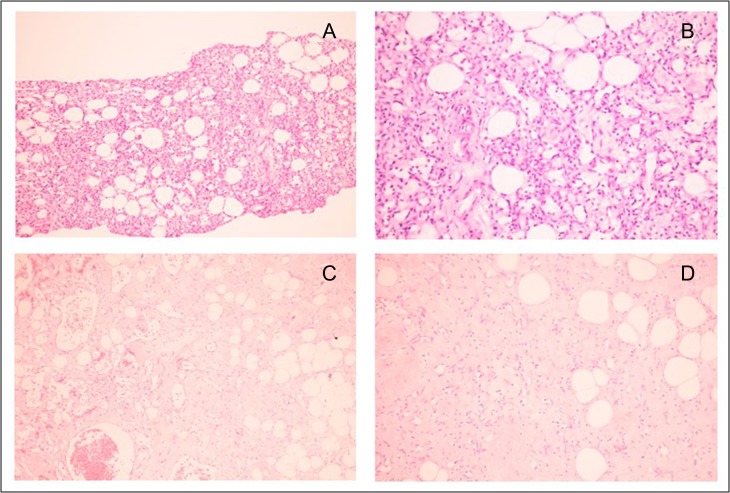
(**A**) Original magnification ×100 and (**B**) original magnification ×400: Core needle biopsy specimen showed spindle-shaped cells proliferation, forming capillary-like vascular channels. Each space calibre is lined by single layer of bland-looking spindle cells, possessing oval hyperchromatic nuclei with fine chromatin. (**C**) Original magnification ×400 and (**D**) original magnification ×400: Wide excision specimen revealed proliferating endothelial cells forming small vessels with proliferative vessels. Some area elicited aggregates of congested vessels.

Ultrasound-guided skin marked wide excision was operated (Fig. 1). The specimen showed area of congested reddish soft tissue mass which contained tumour foci and internal hemorrhag (Fig. 4). The histological result showed proliferating endothelial cells forming small vessels and small areas of elicited aggregation of congested blood vessels. These findings were suggestive of angiomatosis of the breast. No malignant cells which indicated angiosarcoma was demonstrated (Fig. 5).

Postoperatively, patient was instructed to follow up in 6 months for clinical examination and follow up mammogram with ultrasound (Fig. 1). The imaging studies showed complete disappearance of the previous large ill-defined mass at left upper inner quadrant.

## DISCUSSION

Primary vascular tumours of the breast are extremely rare. Angiosarcomas are mostly concerned in accounting for malignant vascular tumour of the breast and comprised <0.1% of all breast malignancy and also the second most common mesenchymal malignancy after phyllodes tumours [1]. They are characterized by rapidly growing, extensively infiltrating atypical cells derived from blood vessels and lining irregular blood-filled spaces. Benign vascular tumours including hemangiomas, angiomatosis, pseudoangiomatous stromal hyperplasia and atypical vascular lesions are clinically indistinct entities. Imaging studies are useful to identify the tumour extent and demonstrated vascular nature. However, the imaging studies cannot totally differentiate between benign and malignant vascular tumour especially angiosarcoma [2]. Therefore, complete surgical excision is usually required [3].

Angiomatosis is very rare benign vascular lesion, mostly found in young women. It consists of large dilated vascular spaces lined by flattened endothelial cells and often involves large area of the breast. Histologic features include the presence of thin to medium sized vascular channels that diffusely involve the breast parenchyma that could mimic low-grade angiosarcoma [1]. Vascular channels are uniformly distributed without the branching lobular pattern of small vascular channels seen in angiosarcoma. The variable degree of cytologic atypia is absent. Local recurrence of angiomatosis has been reported, but metastasis and malignant transformation have not been published [2–4]. Local recurrence probably causes by incomplete excision. Rosen reported two local recurrence of one patient [5].

Mammograms of the angiomatosis demonstrate as an ill-defined or lobulated mass without internal calcifications or axillary lymph node enlargement. Ultrasound has been described as a hypoechoic mass with posterior acoustic shadowing with increased vascularity. Skin thickening of the affected breast may be observed [6, 7]. MRI show an ill-defined mass with low signal intensity on T1WI and high signal intensity on T2WI, strong enhancement and haemorrhagic foci. These findings are undistinguished from angiosarcoma or other vascular lesions which demonstrated the similar signal intensity and kinetic curve enhancement pattern [7, 8].

Treatment is including complete excision or mastectomy for achieving clear margin. Chemotherapy and radiation in adjuvant treatment have not been described [3]. Currently, whole breast irradiation is the standard treatment in breast cancer. While radiation in angiosarcoma is controversial. Hyperfractionated radiotherapy was reported an encouraging outcome [9]. However, external beam radiotherapy is scarcely performed in benign vascular tumour. Portnow *et al.* reviewed the role of external beam radiotherapy that was performed in order to downsizing and symptom relieving in inoperable benign vascular tumours. The radiation dose ranged 36–63 Gy [10]. To our knowledge, this is the first case that preoperative radiation was used to downsizing of primary angiomatosis of the breast. Eventually, breast conserving surgery could be attempted successfully.

In conclusion, this case of primary angiomatosis of the breast is presented as an extremely large infiltrating hypervascular tumour. Imaging studies and histology are inconsistent and cannot distinguish from angiosarcoma. Total removal of breast and skin covering would be offered in terms of conventional treatment. Preoperative radiation could make more favourable outcome in terms of downsizing tumour and achieving breast conservation treatment. Preoperative radiation would be benefit in very large angiomatosis of the breast.
